# Quantification of species-specific meat proteins in cooked and smoked sausages using infusion mass spectrometry

**DOI:** 10.1007/s13197-018-3437-y

**Published:** 2018-09-28

**Authors:** Magdalena Montowska, Anita Spychaj

**Affiliations:** Department of Meat Technology, Poznan University of Life Sciences, Wojska Polskiego 31, 60-624 Poznan, Poland

**Keywords:** Food authenticity, Meat products, Label-free quantification, Peptide markers, Infusion mass spectrometry

## Abstract

Label-free quantification combined with high-resolution infusion-based mass spectrometry (MS) was evaluated to authenticate ‘horse sausages’ made from horse meat and pork. Four types of industrially processed sausages, including cooked horse meat, pork and beef, and their mixtures were analysed. Quantitation and evaluation of the species composition were based on a set of 11 species-specific meat proteins and 14 unique heat-stable peptide markers. Using infusion MS, the highest distinguishing value was found in four proteins, namely, horse myosin-7 (MYH7_HORSE) and horse myoglobin (MYG_HORSE), porcine myosin-4 (MYH4_PIG) and bovine myoglobin (MYG_BOVIN). The limit of detection was 5% (w/w) for pork and beef in the three-component matrix and 1% (w/w) for horse meat. The proteins’ abundance was computed using a peak intensity measurement technique for precursor ions, based on the extracted ion currents/intensities of precursor ions. The procedure enabled discrimination between horse meat, pork and beef proteins, as well as estimation of the relative changes in protein abundance in all the examined samples. Substantial differences in the abundance of specific proteins were obtained from the pure meat samples, three-component mixtures and commercial sausages. The method may be useful in the preliminary screening of protein-rich food samples, aimed at fraud detection and estimation of the overall level of adulteration.

## Introduction

The past success of protein analysis by qualitative mass spectrometry (MS) is tilting in favour of quantitative studies. This trend is due to the increasing availability of high-resolution mass spectrometers and the development of increasingly sophisticated data processing software. Several comprehensive reviews have summarized the latest approaches in the field of quantitative (MS) and its applications in food omics and authenticity research (Neilson et al. 2011; Rodríguez-Suárez and Whetton 2013; Ibáñez et al. 2015; Ortea et al. 2016).

Monitoring the actual proportions of ingredients declared on the label is necessary to recognise the full extent of food adulteration. Some recent MS-based studies investigated meat authenticity issues and established new, unique peptide markers, specific to either or both a given protein and animal species that has led to the distinction between meat and other less valuable additives, such as connective tissue, blood plasma or milk preparations, even in severely processed meat products (Claydon et al. 2015; Montowska et al. 2015; Prandi et al. 2017). Peptidomic profiling of meat belonging to various mammalian species has identified sets of peptide markers unique to beef, pork, lamb and horse meat. In thermally processed samples, myosin, myoglobin, glyceraldehyde-3-phosphate dehydrogenase (GAPDH) and beta-enolase were the main protein sources of peptide markers (von Bargen et al. 2013; Claydon et al. 2015; Watson et al. 2015; Rasinger et al. 2016; Sarah et al. 2016; Montowska et al. 2015). Therefore, these proteins and peptides seem to be the most appropriate for targeted quantitative analysis.

In the context of high-resolution MS-based quantitative techniques, label-based and label-free strategies are the two primary approaches broadly used in protein analysis. Stable isotope labelling of protein and peptides provides superior accuracy, reliability and sensitivity, especially when using an isotope-labelled synthetic peptides strategy (i.e. the AQUA technique) in conjunction with single or multiple reaction monitoring experiments. These peptides need to be homologous to the specific peptides of the targeted proteins. The main disadvantage of this approach is however the cost of isotope labels and, consequently, its low suitability to undertake multi-protein identification in the same run and conduct multi-sample experiments (Mallick and Kuster 2010; Rodríguez-Suárez and Whetton 2013). In these instances, the most economical and simplest solution is to apply the label-free methodology.

Spectral counting and peak area/intensity measurement for precursor ions are the two, leading label-free approaches to tackle quantification of complex protein mixtures. Both are versatile, relatively inexpensive and widely approved as reliable alternatives to labelled procedures, although less accurate. For spectral counting, quantification is based on the total number of distinct spectra acquired from peptides and the number of times the spectra are acquired from a given protein. It works on the principle that a greater number of peptides are obtained from the more abundant proteins present in the sample and, consequently, more spectra are collected and assigned to those proteins compared to less abundant ones (Mallick and Kuster 2010; Rodríguez-Suárez and Whetton 2013). The spectral counting technique has been successfully carried out on complex biological mixtures, such as in the field of cancer proteomics (Zhou et al. 2012) and more recently in meat authentication, to quantify the abundance of meat proteins in various poultry products (Montowska and Fornal 2017). The peak measurement quantification relies on the observation that the electrospray ionization (ESI) signal response is linearly correlated with the concentration of the analysed substance (Rodríguez-Suárez and Whetton 2013). Gallego et al. (2016) quantified the changes in the abundance of the major sarcoplasmic proteins throughout the ham dry-cured process, by measuring the mass spectral peak intensities of the trypsinised proteins from the extracted ion chromatogram. Prandi et al. (2017) established a method to quantify beef and pork in Bolognese sauce, based on the peak areas of two specific peptides originating from the α2-collagen chain. Elsewhere, the peak areas of the marker peptides derived from lupine, pea and soy were applied to calculate the content of selected legume proteins in emulsion-type pork sausages (Hoffmann et al. 2017).

In this paper, high-resolution infusion-based MS/MS and peak intensity measurement techniques were applied to quantify the abundance of pork, beef and horse meat proteins in sausages made predominantly from horse meat. It aimed to evaluate the possibility of protein quantification based on the extracted ion current/intensity (XIC) approach, using the MaxLFQ method implemented in the MaxQuant software (Cox et al. 2014). The software compares intensities across runs and performs XIC-based label-free quantification with high accuracy, particularly when high mass resolution instruments are used. In order to reduce the negative impact of shared peptides (i.e. defined as non-unique or degenerate peptides) on the accuracy of the protein identification and quantitation, consideration was given only to those proteins for which specific peptide markers were assigned.

## Materials and methods

### Materials

Water, acetonitrile, formic acid and methanol LC–MS grade were purchased from Sigma-Aldrich (Schnelldorf, Germany). Ammonium hydrogen carbonate, dithiothreitol (DTT), iodoacetamide (IAA) and all other chemicals were of molecular biology grade and obtained from Sigma-Aldrich. Sequence-grade modified trypsin was bought from Promega GmbH (Mannheim, Germany). Meat samples of cattle, horse and pig (longissimus muscle) and four different types of sausages (S1–S4), labelled as ‘horse sausages’ (coarsely minced, cooked, smoked and semi-dried) were purchased locally. Samples of about 5 g or 5 cm length were cut from raw or processed products and kept at − 80 °C until further protein analysis. Proteins derived from pig (*Sus scrofa*), horse (*Equus caballus*) and cattle (*Bos taurus*) were examined in the present study.

### Preparation of samples

Meat slices of ~ 25 mm thickness were wrapped in aluminium foil and heated in a Rational Combi convection oven (Landsberg am Lech, Germany) at 190 °C until reaching a core temperature of 99 °C (38 min was required), to achieve a high degree of protein denaturation. The core temperature was measured with a 6-point core temperature probe, supplied with the oven. Thin sections of cooked meats and meat products (0.5 g) were rinsed consecutively in ethanol/water, ethanol, methanol/water and milli-Q water, to remove physiological salts, fat and other soluble, low molecular weight contaminants. Washed samples were homogenised in 100 mM of aqueous ammonium bicarbonate using a T25 Ultra-Turrax (IKA Labortechnik, Staufen, Germany) at 9500 rpm for 2 × 20 s, followed by 13,500 rpm for 30 s and then vacuum-dried using a miVac Duo Concentrator (Genevac Ltd, Suffolk, UK).

### Preparation of meat mixtures

Meat mixtures were prepared from washed and dried cooked meats. Samples containing three species (horse, pork and beef) were prepared by weighing respective amounts of the meats, to obtain samples containing equal quantities of two species, spiked with 1% and 5% (w/w) of the third species. A total of 10 mg of the mixture prepared with 1% and 5% (w/w) beef, horse or pork meat was weighed in a 2-mL Eppendorf tube and trypsin-digested.

### Sodium dodecyl sulphate–polyacrylamide gel electrophoresis (SDS–PAGE)

SDS-PAGE was performed to estimate the extent of protein aggregation and degradation. A dried sample of 10 mg was solubilised with lysis buffer (8 M urea, 2 M thiourea, 0.05 mM Tris, 75 mM DTT, 3% SDS, 0.05% bromophenol blue, pH 6.8) and heated at 98 °C for 4 min. Protein concentration was determined using a 2-D Quant kit (GE Healthcare Bio-Sciences, Fairfield, CT, USA). Protein (15 μg) aliquots were loaded onto 15% polyacrylamide gels prepared in a Hoefer SE250 system (GE Healthcare Bio-Sciences). Reference proteins (pre-stained protein M ladder, Thermo Scientific molecular weight standard) was applied. Each gel was electrophoresed a constant current of 20 mA per gel, then stained with Coomassie brilliant blue and scanned (ImageMaster scanner, GE Healthcare Bio-Sciences).

### In-solution trypsin digestion

Dried samples (10 mg) were rehydrated in 100 µL of 50 mM ammonium bicarbonate. The proteins were reduced by 200 mM DTT (56 °C for 1 h) and then alkylated using 200 mM IAA for 30 min in the dark at room temperature. The remaining IAA was quenched by the addition of 200 mM DTT, followed by incubation at room temperature for 30 min. The samples were digested in an ammonium bicarbonate solution containing 0.083 μg/μL of trypsin, at 37 °C, overnight (18 h). The digests were purified by reversed-phase extraction using Sep-Pak C18 Plus cartridges (Waters, Milford, MA, USA). Columns were equilibrated and washed consecutively in solution B (65% acetonitrile, 35% milli-Q water, 0.1% formic acid) and solution A (2% acetonitrile, 98% milli-Q water, 0.1% formic acid), and then the peptide digest sample was added onto the column using a syringe. The sample was washed with solution A and eluted with 2 mL of solution B. Eluted peptides were vacuum-dried using a miVac Duo Concentrator (Genevac Ltd). Before MS analysis, samples were rehydrated in a spray solvent consisting of acetonitrile, water and formic acid (50:50:1 v/v/v).

### Infusion MS/MS analysis

Samples were analysed in chip-based infusion mode via a silicon-based nanoESI microchip. The ion source was a TriVersa NanoMate (Advion, Ithaca, NY, USA) coupled to a Thermo Scientific Q Exactive Hybrid Quadrupole-Orbitrap mass spectrometer (Thermo Fisher Scientific, San Jose, CA, USA) operating in the positive ion ESI detection mode. The NanoMate platform operated at a nanoESI tip voltage of 1.6 kV, with a gas pressure of 2757.9 Pa and a capillary temperature of 190 °C. All the results of the data-dependent analysis (dd-MS^2^ on the top 10 most abundant ions) data were collected in full scan mode with *m/z* range of 50–2000, at 1 microscan, 100 ms maximum injection time and an automatic gain control target of 1e6. Collision-induced dissociation (CID) experiments were performed at a normalised collision energy of 28%. Data were analysed using Xcalibur v. 2.1 software (Thermo Fisher Scientific). For protein and peptide identification, raw files were converted to MASCOT generic format using msconvert ProteoWizard toolkit application (Chambers et al. 2012). The resulting files were searched against the UniProtKB/SwissProt database for the exact matches, using the MASCOT MS/MS ion search program. The database parameters were trypsin enzyme, taxonomy bone vertebrates, one missed cleavage, 1.2 Da peptide mass tolerance, 0.6 Da MS/MS tolerance, carbamidomethylation as fixed modification, oxidation of methionine as variable modification and peptide charges of 2 +, 3 + and 4 +. A decoy search was performed automatically, and the matches and MASCOT scores were evaluated at 1% of the false discovery rate (FDR) for identity and homology threshold. Selected peptides in FASTA format were searched against the NCBInr database using the protein BLAST alignment research tool and blastp algorithm for species and protein specificity.

### MS label-free protein quantification

Label-free protein quantitation was performed using the freely available MaxQuant software and the MaxLFQ algorithms that applied a modified procedure, based on the extracted ion currents/intensities (XICs) of peptides (Cox et al. 2014). All raw files were searched against a set of mammal protein sequences, including isoforms, supplied in FASTA format file and identified earlier in the UniProtKB/SwissProt database search with high MASCOT score and a set of commonly occurring contaminants. The input dataset was processed by the MaxLFQ mode, to generate an output file with the summed up extracted ion current/intensity (XIC) of precursor ions of all isotopic clusters associated with the identified amino acid sequence of a given protein. The MS tolerance was 20 ppm, and the MS/MS fragment ion up to 0.5 Da. MS/MS spectra were filtered to contain maximally ten peaks per 100 mass unit intervals. The intensities of different isotopic peaks in an isotope pattern were summed, and unique plus razor peptides were chosen for the degree of uniqueness of peptides to be included for quantification. This selection, according to Cox et al. (2014), is a good compromise between using as many peptides as possible and only undoubtedly belonging to a given protein. The samples were analysed in two technical replicates.

## Results and discussion

### Identification of proteins and peptide markers

**Fig. 1:**
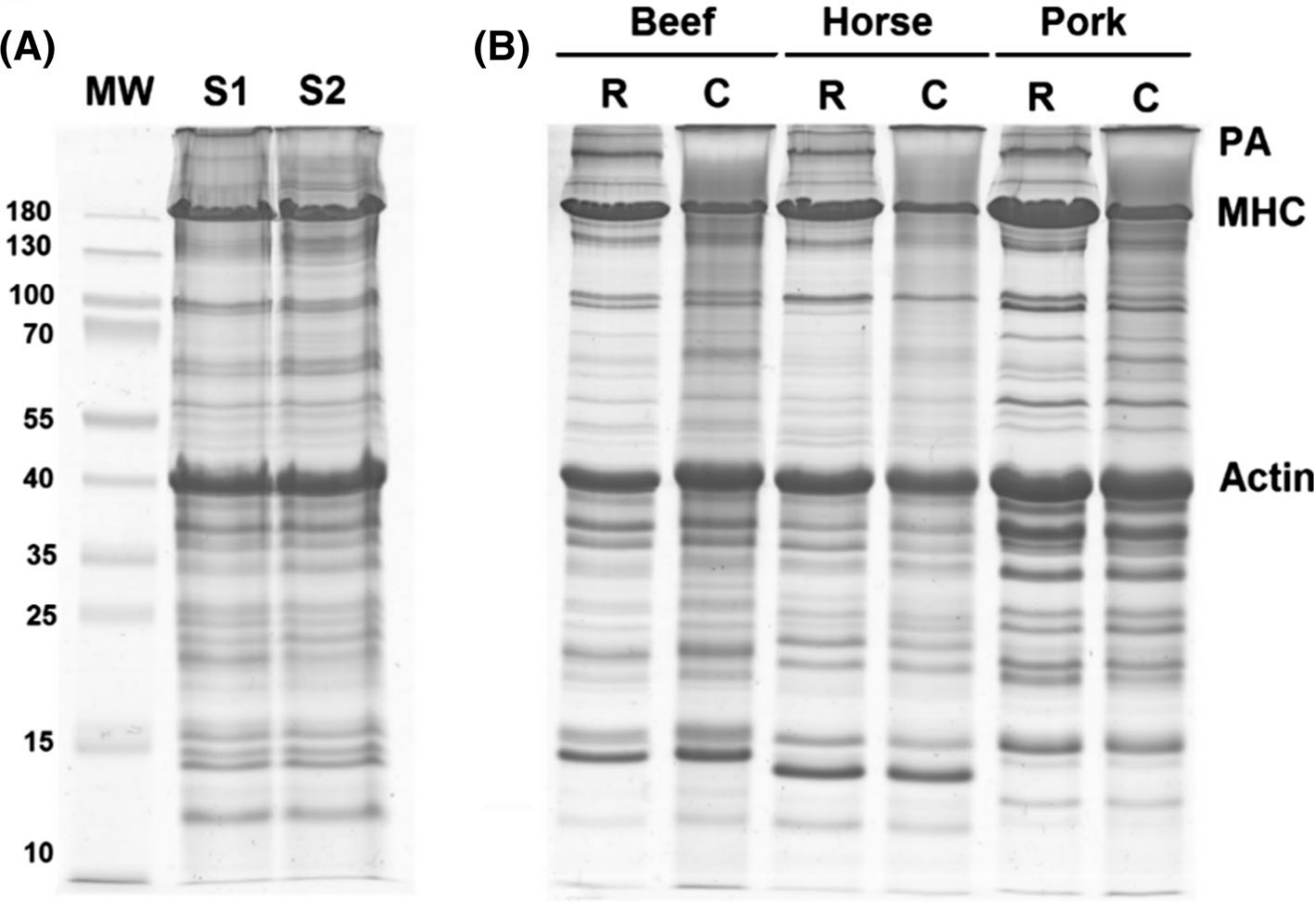
SDS-PAGE protein profile of meat products; **a** commercial horse sausages S1 and S2; **b** raw-R and cooked-C beef, horse meat and pork. Protein bands show considerable protein aggregation (PA) and degradation of the myosin heavy chain (MHC)

Identification of proteins and peptides in food matrices is a challenging task because the result is affected by the technological operations applied during manufacture, as well as the complexity of food matrices. The profiles of proteins extracted from two of the examined sausages and raw and cooked meats are shown in Fig. 1. Thermal treatment is one of the most destructive processes, and this phenomenon was reflected in the electrophoresed gels. On the one hand, substantial degradation of proteins was observed. On the other hand, proteins aggregates were accumulated at the top of the lanes of the processes samples. Differences in the protein composition as well as the impact of processing on protein degradation and aggregation in sausages (Fig. 1a) and cooked beef, pork and horse meat (Fig. 1b), were observed. High molecular weight proteins, including the myosin heavy chains (MHCs), were most affected, while smaller proteins, for example, actin, suffered minimal damage. All the analysed sausages were made of coarsely minced meat, cooked, smoked and semi-dried, and were labelled as ‘horse sausages’ but sold under different product names. The extent of protein damage is mainly related to the type of processing and its conditions (Tornberg 2005; Di Luccia et al. 2015). It is also common knowledge that MS-based studies are negatively affected by heat treatment, due to lower peptide recovery and coverage of protein sequences. Therefore, in this study, for authentication and quantitation purposes, the identification results were carefully evaluated to select species-specific proteins in which the presence of heat-stable peptide markers was confirmed.

**Fig. 2:**
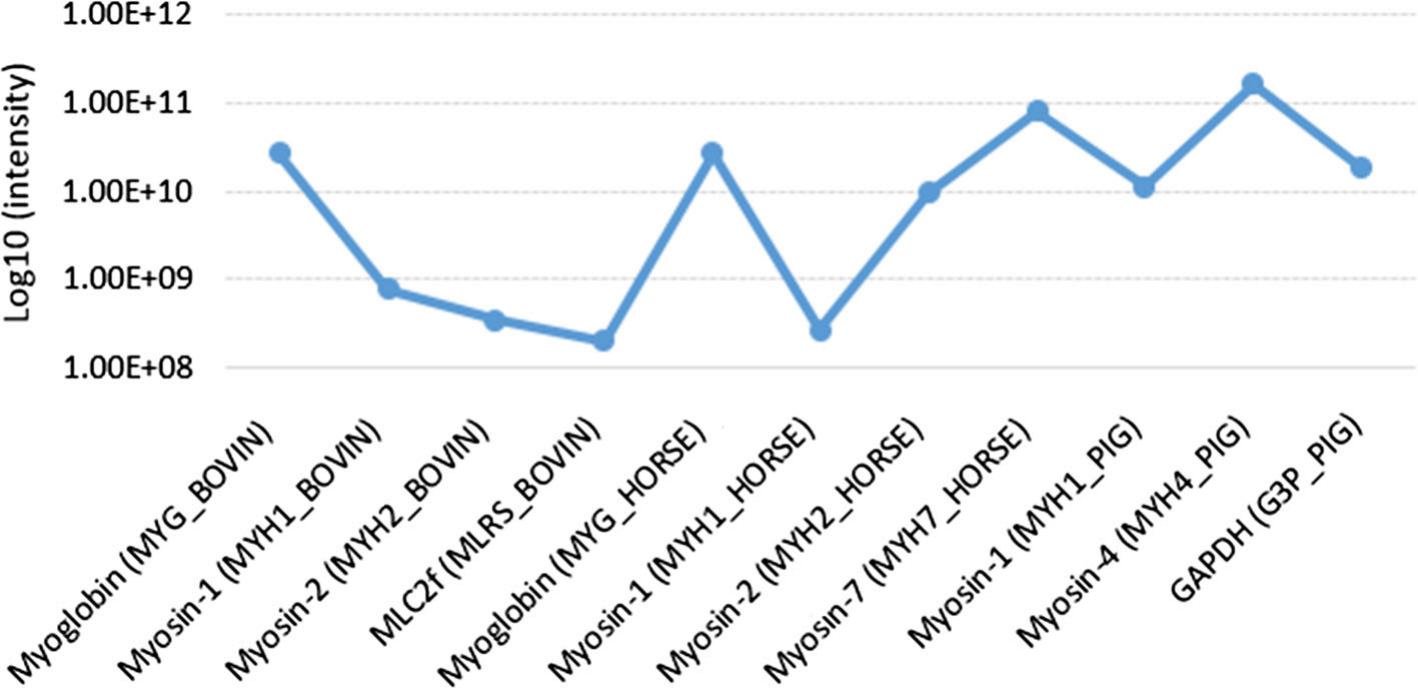
Summed up extracted ion intensity of all isotopic clusters for all tested samples associated with the identified amino acid sequence of selected proteins expressed on a log10 scale

Four different commercial sausages, as well as in-house cooked pork, beef, horse meat and their mixtures, were examined within this study. MS data were collected using a microchip-based infusion mode, with a data-dependent discovery experiment. This approach, as combined with in-solution digestion, was found to be rapid and robust, although less sensitive than LC–MS approach (Montowska and Fornal 2017). Among the most abundant myofibrillar and sarcoplasmic proteins identified at 1% FDR for the identity and homology threshold, 11 turned out to be specific to cattle, horse and pig. These are MHC isoforms, myosin light chain 2 (MLC2), myoglobin and GAPDH. An abundance profile for these proteins across the examined samples was computed by MaxLFQ algorithms, as the cumulative protein intensity across the samples. The values for the selected 11 species-specific proteins ranged from 1.99E + 08 (bovine MLC2f) to 1.65E + 11 (porcine myosin-4) (Fig. 2). Table 1 shows the number of identified peptides, sequence coverages of these proteins, which ranged from 45.1 to 85.9%, as well as species-specific marker peptides. The protein BLAST alignment search tool and blastp algorithm were used for species specificity of the selected tryptic peptides, through all protein sequences stored in the NCBI protein database.

**Table 1:** MaxQuant output scores for species-specific proteins and heat-stable peptide markers unique to cattle, horse and pig identified in cooked meat and sausages

Protein (locus)	Accession number	Peptides^a^	Sequence coverage (%)^b^	Mol. weight (kDa)	Peptide sequence	Mass^c^	Charges^d^	PEP^e^	Score^f^
Myoglobin (MYG_BOVIN)	P02192.3	8	67.5	17.077	HPSDFGADAQAAMSK	1531.6725	2,3	3.93E−44	204.52
Myosin-1 (MYH1_BOVIN)	Q9BE40.2	92	49.7	222.99	TLALLFSGPASGEAEGGPK	1800.9258	2	3.99E−07	79.880
Myosin-2 (MYH2_BOVIN)	Q9BE41.1	87	45.1	223.32	MEIDDLASNVETISK	1663.7975	2	8.07E−02	18.244
MLC2f (MLRS_BOVIN)	Q0P571.1	12	85.9	19.012	EASGPINFTVFLNMFGEK	1999.9713	2	2.34E−02	30.190
Myoglobin (MYG_HORSE)	P68082.2	8	68.2	17.082	GLSDGEWQQVLNVWGK	1814.8951	2	4.17E−17	124.25
					HGTVVLTALGGILK	1377.8344	2,3	6.29E−96	295.78
					VEADIAGHGQEVLIR	1605.8475	2,3	3.81E−44	203.05
Myosin-1 (MYH1_HORSE)	Q8MJV0.1	87	47.9	222.94	VVETMQTMLDAEIR	1634.8008	2	1.81E−10	91.812
					TLALLFSGPASADAEAGGK	1774.9101	2	2.95E−10	95.205
Myosin-2 (MYH2_HORSE)	Q8MJV1.1	93	50.6	222.75	VVETMQTMLDAEIR	1634.8008	2	1.81E−10	91.812
					TLALLFSGAQTADAEAGGVK	1919.0000	2	4.71E−22	157.98
Myosin-7 (MYH7_HORSE)	Q8MJU9.1	95	53.7	223.16	GTLEDQIIEANPALEAFGNAK	2200.1012	2	3.55E−24	151.28
GAPDH (G3P_PIG)	P00355.4	15	72.1	35.836	WGDAGATYVVESTGVFTTMEK	2248.0358	2	2.00E−14	118.05
Myosin-1 (MYH1_PIG)	Q9TV61.1	94	50.6	223.17	SALAHAVQSSR	1125.5891	2	2.02E−09	98.182
Myosin-4 (MYH4_PIG)	Q9TV62.1	95	53.4	223.23	TLAFLFAER	1066.5811	2	1.48E−12	116.52
					SALAHAVQSSR	1125.5891	2	2.02E−09	98.182

^a^The total number of peptide sequences associated with the protein

^b^Percentage of the sequence that is covered by the identified peptides of the protein

^c^Monoisotopic mass of the peptide

^d^All charge states that have been observed

^e^Posterior error probability of the identification. This value essentially operates as a *p* value, where smaller is better

^f^Andromeda score for the best associated MS/MS spectrum

This study confirms that infusion MS-based peptidomic analysis is suitable to authenticate processed products. Some of the peptide markers presented in this paper were also detected in horse and pork samples using LC–MS/MS (von Bargen et al. 2013), which confirms their universal utility as markers of authenticity. In other studies, serum albumin and lactate dehydrogenase peptides were detected as porcine candidate markers in cooked pork (Sarah et al. 2016). Furthermore, several porcine, bovine and ovine peptides were declared potential markers for banned processed proteins in meat and bone meal samples (Marbaix et al. 2016). Additionally, a pork serum albumin antibody-based electrochemical immunosensor designed to detect pork adulteration showed excellent performance in fresh meat but failed in cooked and canned samples, likely due to the destruction of the protein epitopes during heating (Lim and Ahmed 2016). Also, a DNA-based optical fibre genosensor was established as a fast and sensitive method for the detection of minced pork in mixtures with beef, but its application in processed samples was not evaluated (Torelli et al. 2017).

### Limit of detection (LOD)

**Fig. 3:**
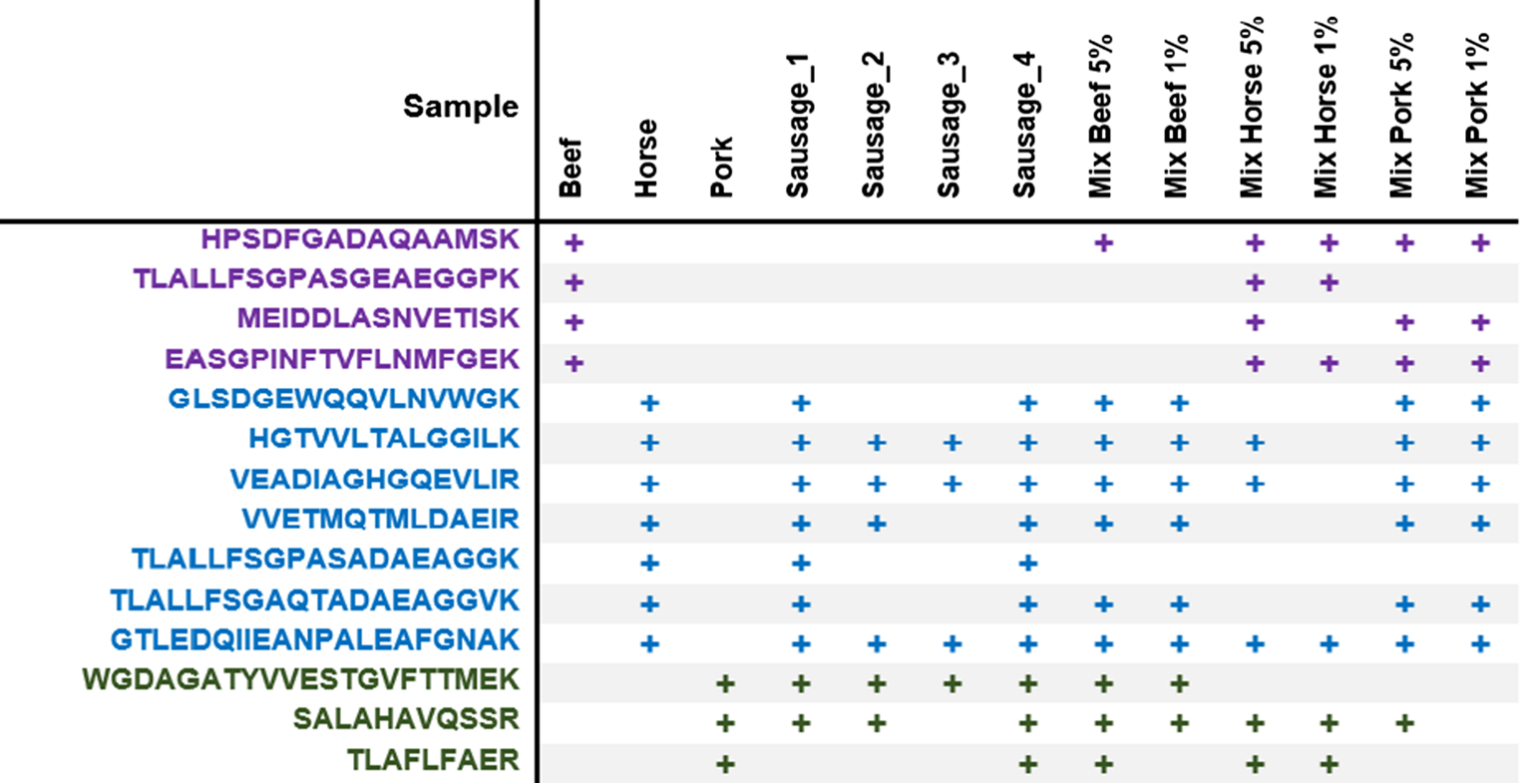
Occurrence of beef, horse and pork peptide markers in single cooked meats, sausages and the three-meat mixtures

In the study, commercial sausages of unknown composition were examined. Therefore, to assess the percentage LOD of the infusion technique, three-component mixtures consisting of cooked beef, pork and horse meat, with 5% and 1% of the third species, were analysed. Single cooked meats served as reference samples. For determination of the LODs, 14 previously selected species-specific peptide markers (Table 1) were monitored in the prepared three-meat samples. The beef, horse and pork peptide markers found in the examined samples, are shown in Fig. 3. Out of four bovine peptides, only one (i.e. myoglobin HPSDFGADAQAAMSK), was detected in the sample containing 5% (w/w) beef in the pork and horse meat matrix. Similarly, for the pork peptides, one peptide SALAHAVQSSR, derived from myosin-1/myosin-4, was detected at 5% (w/w) pork. Improved performance was noted for horse peptide markers. In this instance, three horse peptides, horse myoglobin HGTVVLTALGGILK and VEADIAGHGQEVLIR, and myosin-7 GTLEDQIIEANPALEAFGNAK were sequenced in the sample containing 5% (w/w) horse meat, and the last one was also found in the sample containing 1% (w/w) horse meat in the mixture.

Thus, using the infusion MS/MS method, the LOD for beef and pork was 5% (w/w), whereas horse meat was detected 1% (w/w) in a beef-pork-horse meat mixture. A comparatively more sensitive LC–MS method detected beef, pork, horse and lamb meat at 1% (w/w) in two-component mixtures of fresh meat based on myoglobin-derived peptides (Watson et al. 2015), 0.5% (w/w) horse meat in corned beef with myoglobin peptide markers (Claydon et al. 2015) and 0.5% of pork and beef in thermally processed samples (Li et al. 2018). Moreover, bovine processed animal proteins were detected at 5% (w/w) in pork processed proteins and vegetal feed, based on detection of peptides derived from hemoglobin *α* and heat shock protein *β-*1  (Marbaix et al. 2016).

**Fig. 4:**
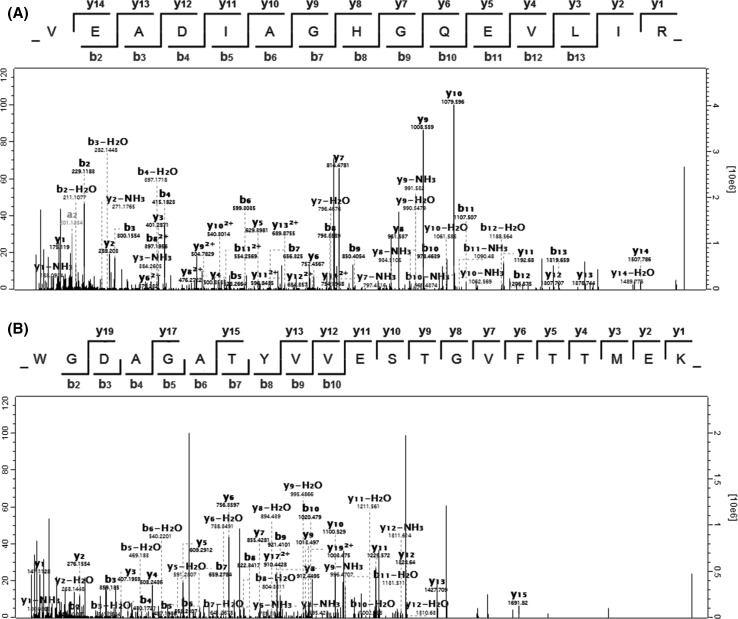
Sequenced mass spectra of horse myoglobin peptide VEADIAGHGQEVLIR (**a**) and pig glyceraldehyde-3-phosphate dehydrogenase peptide WGDAGATYVVESTGVFTTMEK (**b**), obtained from sausage 1 and processed by MaxQuant software

It was established that all the studied four types of commercial sausages sold as “horse sausages” were not pure horse-made products. Pork was also declared on the label but not its percentage. Consequently, an addition of pork was detected in all the examined sausages, due to the presence of pork heat-stable markers (Fig. 3). Figure 4 displays the sequenced mass spectra of horse myoglobin peptide VEADIAGHGQEVLIR (Fig. 4a) and pig GAPDH peptide WGDAGATYVVESTGVFTTMEK (Fig. 4b), obtained from sausage 1 and processed by MaxQuant software. Considering that pork is much cheaper than horse meat, it is likely that a share of horse meat was substituted for pork to reduce the price of the product. The suspicion that the addition of beef could also replace the horse meat has not been confirmed. Therefore, to estimate the share of pork, a further relative quantitative analysis was performed.

### Label-free quantification of meat proteins

Label-free quantification is a much faster and cheaper approach compared to label-based techniques because the expensive labels and extra steps in sample preparation can be avoided. Crucially, moreover, by using this simple analytical workflow dozens, even hundreds of proteins can be analysed in a single MS run. In this work, the label-free quantification was used to evaluate whether it is suitable to estimate differences in protein and species composition of processed products. The quantitation was done by comparing intensities across runs. For this purpose, the MaxQuant tool was applied to all collected MS datasets, allowing a comparison of all the examined samples, i.e. cooked meats, commercial sausages and cooked meat mixtures. The software finds peptides across MS runs and sums the intensities acquired from XICs of peptides assigned to a given protein (Cox et al. 2014). Notably, given that shared peptides in corresponding proteins can have a substantial false-positive impact on quantitative results, only those 11 proteins (Table 1) for which species-specific peptide markers were detected, were taken to further evaluate changes in the protein abundance of a given species per sample.

**Fig. 5:**
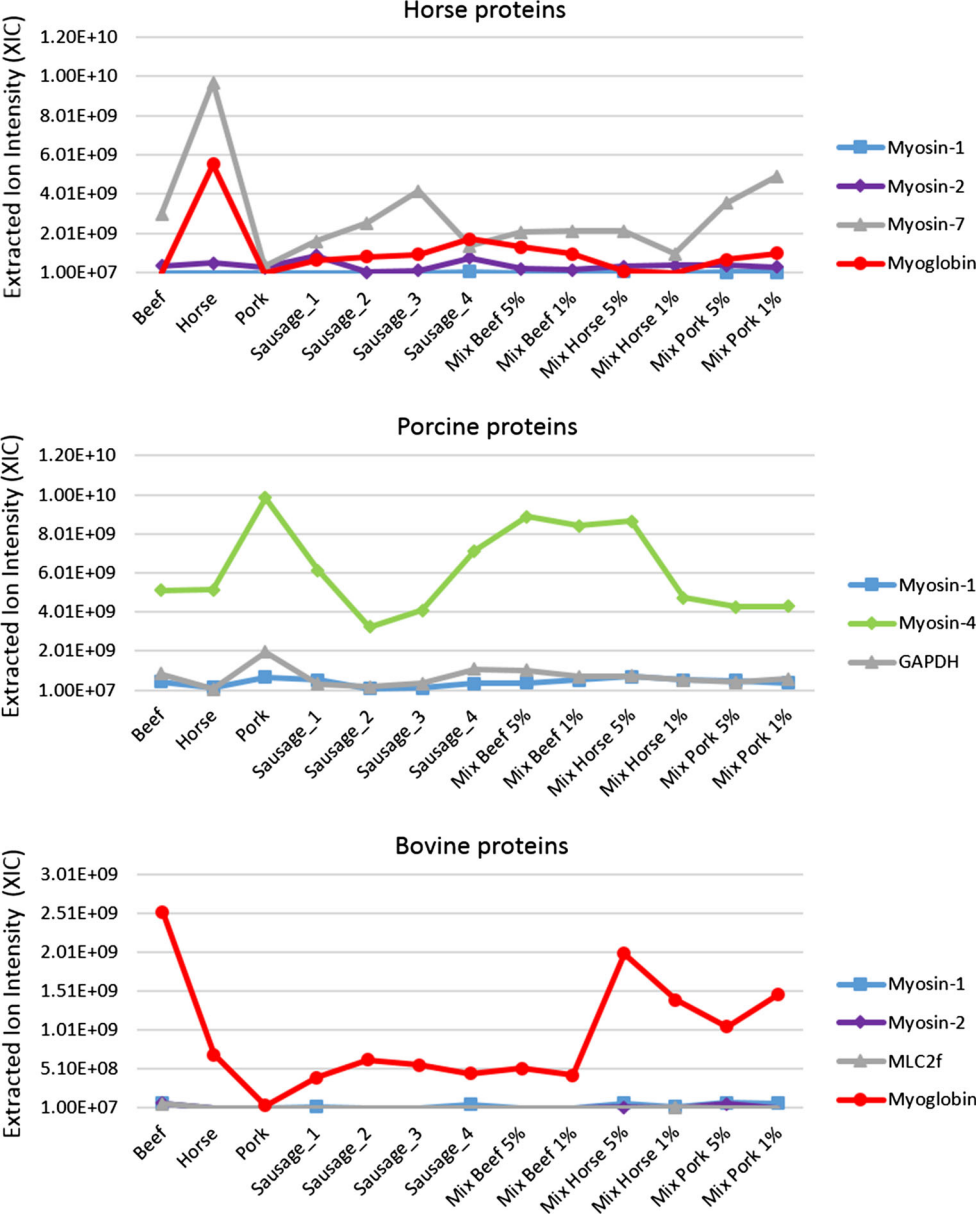
Changes in the protein content of specific horse, porcine and bovine proteins among samples. Results of the label-free quantification of specific proteins were obtained from the MaxQuant proteomic platform and expressed as the summed extracted ion current (XIC) of all isotopic clusters associated with the identified amino acid sequence of a given protein

Figure 5 presents the label-free relative quantification data regarding the specific horse, porcine and bovine proteins among the samples, calculated by the MaxQuant proteomic platform. The three graphs show substantial protein differences between species per sample. Overall, significantly higher values across all samples were observed for horse myosin-7 (MYH7_HORSE) and myoglobin (MYG_HORSE), porcine myosin-4 (MYH4_PIG) and bovine myoglobin (MYG_BOVIN). These proteins, assigned to these three species, generated the highest intensity values due to their high abundance in the examined samples and likely good ionisation susceptibility, which could have enhanced the efficiency of the direct infusion nanoESI.

For the reference samples that contained horse, pork or beef, exclusively, the highest values of all samples were obtained for the proteins of these species (Fig. 5). In the case of horse proteins, for “horse sausages” products with unknown shares of the meat components, higher abundances of horse myosin-7 were observed for sausages 2 and 3 relative to sausages 1 and 4. Conversely, an inverse proportion of pork proteins was obtained, with higher intensities in sausages 1 and 4 than sausages 2 and 3. All these samples were made from horse meat and pork. However, the results suggested that sausages 1 and 4 contained less horse meat and more pork, but the opposite was observed for sausages 2 and 3. These data confirm that a share of horse meat was substituted for pork and the sausages 1 and 4 contain about twice the portion of pork compared to the other two sausages. In the absence of horse meat, pork and beef, or for samples containing small quantities, the lowest levels of specific proteins were acquired. For instance, the abundances of beef proteins in all sausages where they were not detected were at the lowest and equal levels, whereas, the highest peaks for beef myoglobin and beef myosins occurred in pure beef samples and mixtures containing about 50% beef. Typically, the relation between the amounts of horse meat, pork and beef across the samples was correct. Nonetheless, in mixtures, the results were sometimes overestimated, probably due to the presence of large quantities of shared peptides (i.e. non-unique or degenerate peptides), which could have affected the accuracy of the proteins abundance and their quantification.

The results presented in this article prove that label-free quantification combined with infusion MS can be implemented for authenticating complex meat products. This strategy is much more straightforward, cost-effective and simpler than other label-free methods using liquid chromatography coupled with MS, which have been reported in the literature so far (Gallego et al. 2016; Prandi et al. 2017). Besides, when using our approach only a high-resolution mass spectrometer and freely available software are required. This inexpensive strategy can be used for rapid screening of meat products and potentially other protein-rich products, targeting at fraud detection. However, considering the presented method is suitable for relative quantification, absolute quantification using labelled peptides should be implemented to estimate the quantity of adulteration of particular components accurately.

## Conclusion

Label-free quantification combined with infusion high-resolution MS was implemented for authenticating complex meat products. The procedure enabled discrimination between horse meat, pork and beef proteins, as well as estimation of relative changes in protein abundance derived from these three species in meat mixtures and four types of differently processed commercial sausages. Infusion MS could detect meat-specific peptides in cooked meat mixtures down to 5% (w/w) pork and beef and 1% (w/w) horse meat. Quantification and evaluation of the species composition in all the examined samples were based on a set of 11 species-specific proteins, which turned out to be the source of 14 unique peptides, detectable using this approach. Substantial differences in the abundance of specific proteins were obtained from among the pure meat samples, three-component mixtures and industrially processed sausages. The procedure may be useful in the preliminary screening of protein-rich food samples, aimed at detection of adulteration. Although the method has discriminatory power, it can be used for relative quantification only and, thus, absolute quantification techniques with labelled peptides should be applied to precisely establish the extent of suspected adulteration.
